# Blocking autophagy with chloroquine aggravates lipid accumulation and reduces intracellular energy synthesis in hepatocellular carcinoma cells, both contributing to its anti-proliferative effect

**DOI:** 10.1007/s00432-022-04074-2

**Published:** 2022-06-13

**Authors:** Fengming Xu, Hans-Michael Tautenhahn, Olaf Dirsch, Uta Dahmen

**Affiliations:** 1 Department of General, Visceral and Vascular Surgery, Jena University Hospital, 07747 Jena, Germany; 2 Else Kröner Graduate School for Medical Students “JSAM”, Jena University Hospital, 07747 Jena, Germany; 3 Else Kröner Research Schools for Physicians “AntiAge”, Jena University Hospital, 07747 Jena, Germany; 4 Institute of Pathology, Klinikum Chemnitz gGmbH, 09111 Chemnitz, Germany

**Keywords:** Chloroquine, Autophagy, Energy, Lipid, Liver, Cancer

## Abstract

**Purpose:**

The autophagy inhibitor chloroquine enhances the effect of targeted therapy using tyrosine kinase inhibitor in liver cancer. We would like to further understand the specific mechanism by which chloroquine inhibits the proliferation of tumor cells.

**Methods:**

We used a human hepatocarcinoma cell line (HepG2) as cell culture model. In contrast to the control groups (treated only with complete medium), cells in experimental groups were treated either with complete medium + 40 ng/ml Hepatocyte growth factor (HGF), or with complete medium + 60 μM chloroquine or with complete medium + 40 ng/ml HGF + 60 μM chloroquine for 24 h. Cell number and ATP content were investigated using spectrophotometric assays. Cell proliferation and apoptosis were detected by immunohistochemistry. Cell morphological alterations were examined by Giemsa and H&E staining. Cellular lipid content was determined by Oil Red O staining and Triglyceride quantification assay. Autophagy-related proteins (LC3B and p62) and hepatocyte proliferation-related protein (S6K1) were examined using western blot. The autophagic flux of cells was assessed by mRFP-EGFP-LC3 transfection assay.

**Results:**

We found that chloroquine inhibited the proliferation of HepG2 cells, as evidenced by a decrease in cellular ATP content, Ki-67 and S6K1 protein expression and a reduction in cell number. This finding was associated with an increase in lipid content. As expected, chloroquine inhibited autophagy of HepG2 cells, as evidenced by the accumulation of LC3B-II and the significant upregulation of p62. mRFP-EGFP-LC3 transfection assay showed that indeed chloroquine blocked the autophagic flux in HepG2 cells.

**Conclusion:**

Chloroquine impaired proliferation of HepG2 cells might be due to intracellular accumulation of lipids and inhibition of energy synthesis.

## Introduction

Primary liver cancer is one of the most common cancers in the world. According to a 2020 global cancer statistics, liver cancer has the seventh-highest incidence among 36 common cancers and the second-highest mortality. The incidence of hepatocellular carcinoma is increasing in elderly patients (Brunot et al. 2016; Sung et al. 2021).

Surgery is the best curative option for hepatic tumors. However, in case of unresectable tumors, systemic treatment, either in neoadjuvant or palliative intention, is applied. Tyrosine kinase inhibitors (TKIs) are frequently used but may cause adverse effects such as heart failure. Furthermore, TKI can not necessarily be applied for the long term due to the development of drug resistance (Hartmann et al. 2009; Tang et al. 2020; Wu and Shemisa 2017). Therefore, adding other drugs to the systemic treatment protocols could be of benefit.

Evidence is accumulating that modulation of autophagy could be an interesting option (Huang et al. 2018). Autophagy is an important mechanism for recycling intracellular components in eukaryotic cells. During autophagy, defective organelles or misfolded proteins are degraded into basic components for later reuse via the lysosome-dependent pathway. Autophagy plays an active role in maintaining cellular energy homeostasis and controlling organelle quality (Glick et al. 2010).

Chloroquine is a well-known autophagy inhibitor. In the last 50 years, it was mainly used for the treatment of malaria and connective tissue diseases such as systemic lupus. Its good clinical safety profile results in a low incidence of adverse events even in case of long-term administration for up to several years (Savarino et al. 2003).

Chloroquine has interesting biochemical properties. It is a lysosomotropic compound that increases lysosomal pH, thereby inhibiting the activity of lysosomal hydrolases and preventing the fusion of autophagosomes and lysosomes (Dunmore et al. 2013; Fedele et al. 2020; Ye et al. 2016). Due to these properties, chloroquine interferes with the late phase in the autophagy process.

Moreover, evidence has accumulated that chloroquine can inhibit the growth of a variety of tumor cells, such as lung cancer and colon cancer cells and also hepatocellular carcinoma cells (HCCs) (Fan et al. 2006; Hu et al. 2016; Zheng et al. 2009). However, the specific mechanism inhibiting proliferation of HCCs remains unclear.

We would like to further explore the anti-proliferative effect to provide new ideas for safer and more effective systemic therapy for hepatocellular carcinoma.

Division and growth of hepatocytes require ample energy supply, as also pointed out in our previous literature reviews (Alexandrino et al. 2018; Xu et al. 2020). Degradation of damaged organelles such as dysfunctional mitochondria via autophagy is needed to promote ATP production and thereby support cell proliferation (Mizushima 2007; Palikaras et al. 2018; Pickles et al. 2018).

HCCs have higher metabolic activity compared to normal hepatocytes (Zarrinpar 2017). Fatty acids are an important fuel for the generation of ATP. Autophagy can degrade lipid droplets into free fatty acids (FFAs) by acidic lipolysis (Liu and Czaja 2013; Zechner et al. 2017). The complete oxidation of one palmitic acid (a common saturated fatty acid) molecule produces 129 ATP molecules (Bhagavan and Bhagavan 2002). Lipid droplets stored in HCCs may provide a potential source of energy for cell proliferation.

We raise the hypothesis that chloroquine may inhibit HCCs proliferation by causing lipid accumulation in HCCs resulting in a reduction of intracellular ATP generation, both due to the inhibition of autophagy.

## Materials and methods

### Experimental design

We used the human hepatocellular carcinoma cell line (HepG2) as a study model.

Control group was left untreated. The experimental groups were subjected to treatment with Hepatocyte growth factor (HGF) or chloroquine (CQ) or the combination of HGF and CQ (HCQ) for 24 h. HGF is known to be a potent hepatocyte mitogen, and it was used here as a positive control.

Cell density and morphology as well as the rate of proliferating, respectively, apoptotic cells were investigated using a cytoblock technique. Cell number (CCK-8) and ATP content were investigated using spectrophotometric assays. Lipid accumulation was detected by Oil Red O staining and Triglyceride quantification assay. Autophagy-related proteins (LC3B, p62) and hepatocyte proliferation-related protein (S6K1) were examined using western blot. Autophagic flux was detected by mRFP-EGFP-LC3 plasmid-transfection assay. Each experiment was repeated three times.

### Materials

Chemicals and solutions were mainly purchased from Sigma-Aldrich and ThermoFisher. E.g., Hepatocyte growth factor (Sigma-Aldrich, #H9661), Chloroquine (Sigma-Aldrich, #C6628), Fetal Bovine Serum (FBS) (Sigma-Aldrich, #7524), Dulbecco’s Modified Eagle Medium (DMEM) (Gibco, #41965-039) and Penicillin/Streptomycin (C.C.Pro, Z-13-M).

### Cell culture

We obtained the HepG2 cells from a previous cooperation partner of the lab (Dr. Kaufmann). HepG2 cells were cultured in 75cm^2^ cell culture flasks (Greiner bio-one, #108759) with DMEM supplemented with 10% FBS and 1% penicillin and streptomycin (P/S). All cell cultures were maintained at 37 °C in a cell incubator with 5% CO_2_ and 95% air. A hemocytometer was used for cell counting. HepG2 cells were treated with complete DMEM (control) or with complete DMEM + 40 ng/ml Hepatocyte growth factor (HGF), or with complete DMEM + 60 μM Chloroquine (CQ) or with complete DMEM + 40 ng/ml HGF + 60 μM CQ for 24 h.

### Cell counting kit-8 assay

The cell number was determined using a cell counting kit 8 (Abcam, #ab228554). Following the instruction of the manufacturer, HepG2 cells (5 × 10^3^ cells/well) were cultured for 24 h in triplicates using a 96-well tissue culture plate with 100 μL/well of complete DMEM or medium containing 40 ng/ml HGF, 60 μM CQ and 40 ng/ml HGF + 60 μM CQ, respectively. Next, 10 µl of WST-8 solution was added to each well and incubated for another 4 h at 37 °C in the dark. The optical density was read immediately using a spectrophotometric microtiter plate reader (Synergy LX) set at a wavelength of 460 nm.

### Immunohistochemistry

The cell proliferation and apoptosis were detected by immunohistochemistry. The cytoblock technique was used for histology to assess cell number and morphology as well as for immunohistochemistry to determine the rate of cells undergoing proliferation or apoptosis using antibodies targeting the Ki-67, respectively, Caspase-3.

At the end of the cell culture observation time, cells were trypsinized and subjected to alcoholic fixation into CytoRich Red solution for at least 24 h. Cells were centrifuged and the pellet was embedded in a gel, followed by paraffin embedding. Sections of 4 µm thickness were prepared. Antigen retrieval was performed according to the requirements of the antibody specification. The sections were incubated with peroxidase blocking solution (Dako, #SM801) for 5 min to quench endogenous peroxidase activity. Primary antibodies mouse anti-Ki-67 antigen clone MIB-1 (Ki-67; Ready to use, Dako, #IR626) and rabbit anti-Caspase-3 cleaved (Caspase-3; 1:50, Zytomed Systems, #RBK009-05) were applied to the sections and were incubated at room temperature (RT) for 20 (Ki-67)/30 (cleaved Caspase-3) minutes. After washing the sections using washing buffer, the second antibody (Dako, #SM802) was applied. The sections were incubated at RT for 20 min. After washing the sections using washing buffer, DAB solution (Dako, #SM803) was applied to the sections and incubated at RT for 10 min. After washing the sections using washing buffer, hematoxylin was applied to the sections followed by 5 min incubation at RT.  Sections were scanned by the Hamamatsu Slide Scanner (Nanozoomer XR). Quantitative analysis of sections using HistoCAD VirtualLiver (Fraunhofer Mevis, Bremen, Germany). The HistoCAD VirtualLiver used machine learning techniques to recognize cell positive and negative patterns.

### Giemsa staining

The cell morphological changes were detected by Giemsa staining. HepG2 cells were fixed in CytoRich Red solution for at least 24 h. Sections of 4 µm thickness were prepared. Sections were first stained with Giemsa solution at RT for 2 h. After washing the sections using distilled water, acetic acid solution was applied to the sections and incubated at RT for 1 min. Sections were scanned by the Hamamatsu Slide Scanner (Nanozoomer XR).

### Hematoxylin–eosin staining

The routine histological examination was performed by Hematoxylin–eosin (H&E) staining. HepG2 cells were fixed in CytoRich Red solution for at least 24 h. Sections of 4 µm thickness were prepared. Sections were first stained with hematoxylin at RT for 5 min. After washing the sections using distilled water, eosin was applied to the sections and incubated at RT for 1 min. Sections were scanned by the Hamamatsu Slide Scanner (Nanozoomer XR).

### Oil red O staining

The lipid staining was performed using an Oil Red O staining kit (Sigma-Aldrich, #MAK-194). HepG2 cells (5 × 10^4^ cells/well) were plated triplicates in a 24-well plate with 500 μL/well of complete DMEM. Cells were treated with complete DMEM or medium containing 40 ng/ml HGF, 60 μM CQ and 40 ng/ml HGF + 60 μM CQ, respectively, for 24 h. Then, cells were fixed in 10% formalin for 1 h, the formalin was discarded and the cells were washed three times with ultrapure water. The cells were incubated with 60% isopropanol for 5 min. The 60% isopropanol was discarded and the cells were incubated with Oil Red O Working Solution for 20 min. The Oil Red O Working Solution was discarded and the cells were washed three times with ultrapure water. Next, hematoxylin was added to the cells and incubated for 1 min. The hematoxylin was discarded and the cells were washed three times with ultrapure water. Cells were covered with 500 µl ultrapure water. Visualization of Oil Red O staining in HepG2 cells was performed using a Leica optical microscope (DM IRB).

### Triglyceride quantification assay

The cellular triglyceride (TG) content was measured using a triglyceride quantification kit (Abcam, #ab65336). Triglyceride standard curves were established using the triglyceride standards provided in the kit. 1 × 10^7^ cells were harvested and lysed to prepare cells lysates. 50 μl reaction mixture was prepared for each reaction. The triglyceride standard wells (50 μL/well), sample wells (50 μL/well) and sample background control wells (50 μL/well) were set up in a 96-well tissue culture plate. 2 μL of lipase was added to the standard wells and sample wells, 2 μL of triglyceride assay buffer was added to the sample background control wells and incubated at RT in the dark for 20 min. 50 μl reaction mixture was added to each standard well, sample well and sample background control well and incubated at RT darkroom for 60 min. The output optical density was read immediately using a microplate reader (Synergy LX) at the wavelength of 570 nm.

### ATP detection assay

The ATP assay was performed using an ATP detection kit (Abcam, #ab83355). ATP standard curves were established using the ATP standards provided in the kit. 3 × 10^6^ cells were harvested and lysed to prepare cells lysates. Deproteinizing Sample Preparation Kit (Abcam, #ab204708) was used to remove superfluous enzymes. 50 μl reaction mixture and background mixture were prepared for each reaction. The ATP standard wells (50 μL/well), sample wells (50 μL/well) and sample background control wells (50 μL/well) were set up in the 96-well tissue culture plate. 50 μl reaction mixture was added to each standard well and sample well, 50 μL background reaction mixture was added to each sample background control well and incubated at RT in the dark for 30 min. The optical density was read immediately using a microplate reader (Synergy LX) at the wavelength of 570 nm.

### Western blotting

Cells were lysed in the RIPA buffer (Sigma, #R0278) containing a protease and phosphatase inhibitor cocktail (Thermo Scientific, #78442). The concentration of total proteins was measured using the BCA protein assay kit (Thermo Scientific, #23225) and a Synergy LX Multi-Mode Microplate Reader. Equal amounts (20 µg) of protein were denatured with Laemmli sample buffer. Proteins were separated in electrophoresis and transferred to polyvinylidene fluoride (PVDF) membranes. The membranes were washed with Tris-buffered saline with tween 20 (TBST) and blocked with 5% BSA-TBST buffer. Primary antibodies rabbit anti-light chain 3 (LC3; 1:1000, Abcam, #ab48394), rabbit anti-SQSTM1 (p62; 1:10,000, Abcam, #ab109012), rabbit anti-S6K1 (1:2000, Abcam, #ab32359) and rabbit anti-glyceraldehyde-3-phosphate dehydrogenase (GAPDH; 1:15,000, Abcam, #ab181602) were applied to the membranes and were incubated at 4 °C overnight. After washing the membranes using TBST, the second antibody (polyclonal goat antibody to rabbit IgG; 1:10,000, Abcam, #ab6721) was applied. The PVDF membranes were incubated at 37 °C for 1 h. After being probed using enhanced chemiluminescence western blotting detection reagents (GE Healthcare, #RPN2209), signals were visualized using Fusion FX7 (Labtech International Ltd, Heathfield, United Kingdom). Quantitative analysis of western blotting results was performed using the software Image J.

### mRFP-EGFP-LC3 plasmid transfection

The HepG2 cells were transfected with the mRFP-EGFP-LC3 (ptfLC3) plasmid (Addgene plasmid, #21074, ptfLC3 was a gift from Prof. Tamotsu Yoshimori) using Lipofectamine 2000 kit (Lipofectamine, #11668019) according to the instruction (Kimura et al. 2007). Briefly, we isolated a single colony of bacteria from the Lysogeny broth (LB) agar plate, followed by placing the colony into the LB Broth medium (Sigma, #L2542) for further expansion. Next, a plasmid DNA extraction kit (QIAGEN Plasmid Midi Kit, #12143) was used to isolate the plasmid DNA. Plasmid DNA was transfected into HepG2 cells using the Lipofectamine 2000 kit. 24 h after transfection, cells were cultured in complete medium or medium containing 40 ng/ml HGF, 60 μM CQ and 40 ng/ml HGF + 60 μM CQ for 24 h, respectively, at 37 °C in a cell incubator with 5% CO_2_ and 95% air. Visualization of transfected HepG2 cells was performed using a Zeiss fluorescence microscope (AX10).

### Statistical analysis

Data analysis was performed with the statistical software SPSS 25.0 and SigmaPlot 13.0 (Statcon, Witzenhausen, Germany). The differences between groups were assessed using the one-way ANOVA test with LSD post hoc analysis (Data follow normal distribution or approximately normal distribution and homogeneity of variance) or Kruskal–Wallis test (Data do not follow normal distribution or homogeneity of variance). We pooled the data from three rounds of repeated experiments for statistical analysis. Statistical differences were considered significant when *P* < 0.05.

## Results

### Chloroquine significantly reduced the number of HepG2 cells

**Fig. 1:**
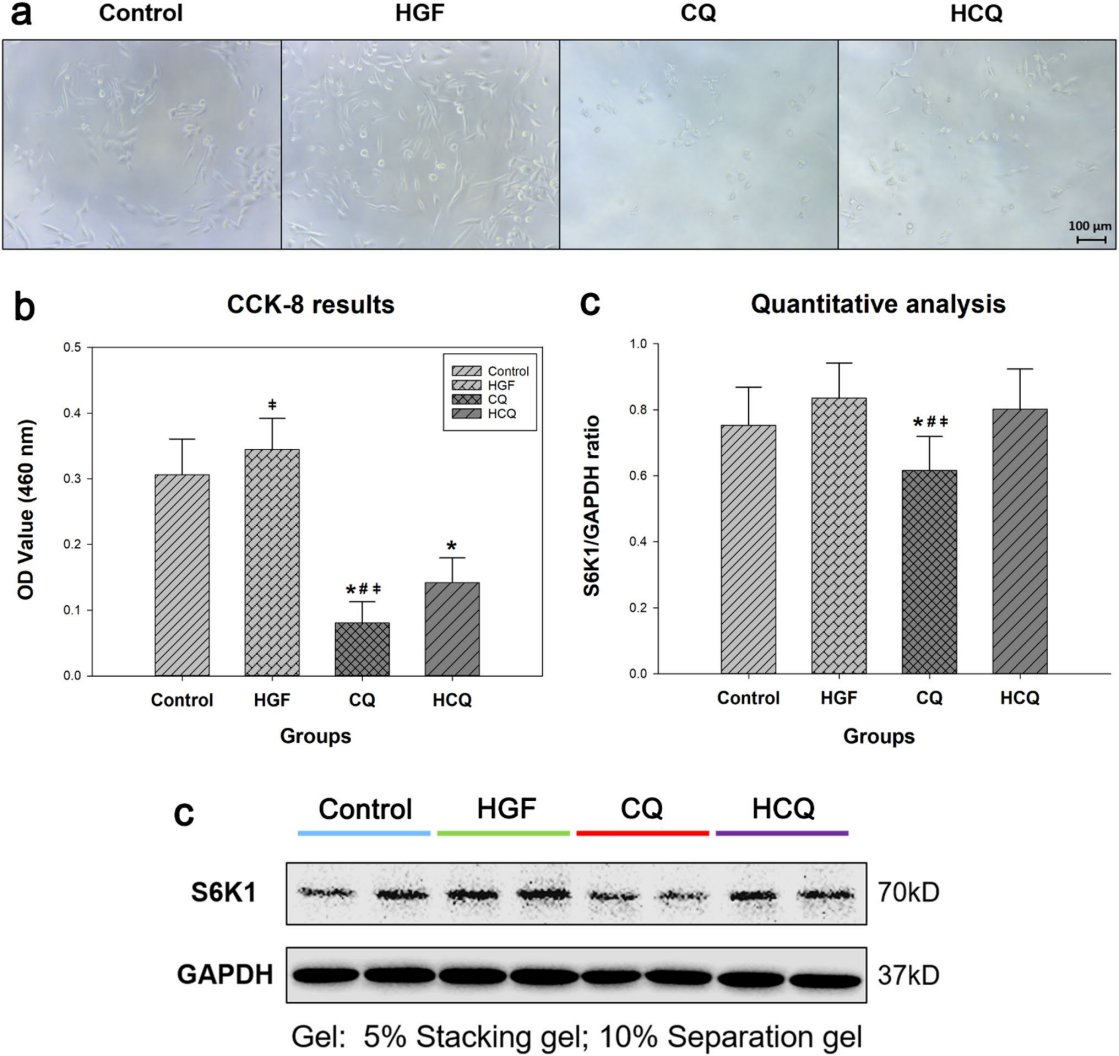
Chloroquine effectively reduced the number of HepG2 cells. **a** HepG2 cells were treated with different drugs for 24 h followed by microscopical assessment of confluence (Leica DM IRB microscopy, magnification: 100X). **b** HepG2 cell number was measured by CCK-8 assay. **c** The hepatocyte proliferation-associated protein S6K1 of HepG2 cells was determined by western blot, GAPDH was used as control. (*represents significant difference compared to control, # represents significant difference compared to HGF, ǂ represents significant difference compared to HCQ)

First, we wanted to verify the effect of chloroquine on HepG2 cells in terms of proliferation. Before starting cell quantification assay with CCK-8, we observed cell confluence microscopically (Leica optical microscope DM IRB). It was very obvious that CQ treatment resulted in a less confluent cell layer, compared to the control group (Fig. 1a) suggesting a reduced proliferation rate. In contrast, HGF resulted in a more confluent cell layer indicative of a higher proliferation rate.

**Fig. 2:**
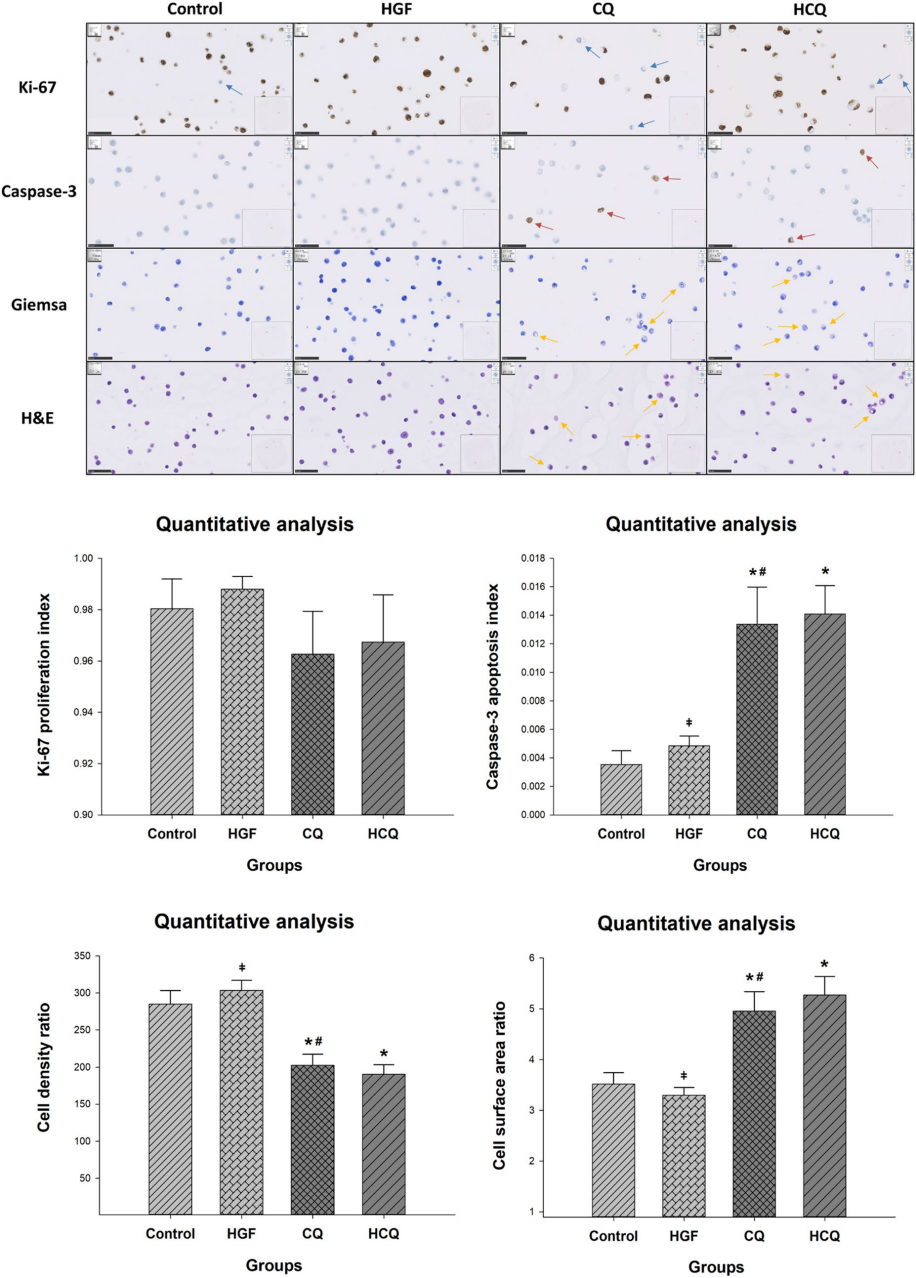
Chloroquine inhibited the proliferation of HepG2 cells and caused significant apoptosis. HepG2 cells were treated with different drugs for 24 h and the cell proliferation and apoptosis were determined by immunohistochemistry; The cell morphological alterations were detected by Giemsa and H&E staining (*represents significant difference compared to control, # represents significant difference compared to HGF, ǂ represents significant difference compared to HCQ)

This impression was further substantiated when determining the cell number in the cytoblock sections. We quantified the relative cell density (cell number/unit area) using two differently stained sections from three cytoblocks, each representing an independent cell culture experiment. The cell density was increased in the HGF group and reduced in the CQ group compared to the control (Fig. 2), both indicative of the proliferation inducing, respectively, inhibiting effect of the two drugs.

Using another independent assay reflecting the cell number did support this interpretation. The CCK-8 assay revealed (Fig. 1b) that treatment with CQ for 24 h resulted in a significantly lower signal (decrease of about 74% OD 460 index) compared to control (*P* < 0.01), indicating a lower cell number either resulting from a higher death or a lower proliferation rate. In contrast, HGF-treated groups showed higher signals by about 13%, but the difference was not significant. Compared with the HGF group, the OD 460 index of the HCQ group decreased by approximately 59%, which indicated a significant inhibition of proliferation (*P* < 0.01).

To confirm that the difference in cell number was indeed the result of decreased proliferation, we stained the cells for Ki-67 and performed a western blot for the proliferation-associated Ribosomal protein S6 kinase beta-1 (S6K1).

Ki-67 is one of the most widely used markers to measure cell proliferation. Our immunohistochemical results showed that CQ down-regulated Ki-67 protein expression in HepG2 cells compared to controls, while HGF upregulated Ki-67 expression in the cells, but none of these differences reached statistical significance (Fig. 2, blue arrows indicate Ki-67 staining-negative cells).

The protein S6K1 is one of the important regulators of hepatocyte proliferation. (Espeillac et al. 2011) demonstrated that proliferation of hepatocytes after hepatectomy requires the activation of S6K1. Our results showed (Fig. 1c) that CQ significantly inhibited S6K1 expression in HepG2 cells compared to the control group (*P* < 0.05). The S6K1/GAPDH ratio showed a decrease by approximately 18%, suggesting the inhibition of cell proliferation. HGF upregulated S6K1 expression in HepG2 cells by about 11%, but the difference was not significant. Compared with the HGF group, the S6K1/GAPDH ratio of the HCQ group decreased by about 4%, albeit not reaching statistical significance. We think that the reduction in cell number observed in the CQ group was, at least partially due to a reduced proliferation.

Since differences in cell numbers can not only be the result of decreased proliferation but also due to differences in cell death, we investigated apoptosis as well. Using the Caspase-3 staining from the cytoblock sections, we quantified the rate of cells undergoing apoptosis (Fig. 2). Although the overall rate was rather low, we observed a three-fold higher rate in the CQ-treated group (1.3%) compared to the control and HGF group (0.4 and 0.5%, *P* < 0.01). This observation suggests that CQ treatment also induced apoptosis.

Besides, Giemsa and H&E staining results showed that the HepG2 cell size seemed to be increased in the CQ and HCQ groups compared to the control group. Therefore, we calculated the cell surface area ratio. The results showed (Fig. 2) that CQ and HCQ treatment resulted in a significant increase in the surface area ratio of HepG2 cells (*P* < 0.01), which may be related to the accumulation of intracellular lipids.

### Chloroquine aggravated lipid accumulation in HepG2 cells

Both histological stainings, Giemsa as well as H&E, revealed remarkable difference between the cells in the chloroquine-treated groups compared to the control group. CQ-treated cells appeared larger due to an accumulation of small intracytoplasmic vesicles (Fig. 2). This prompted a morphometric assessment calculating the cell surface area ratio. Indeed, the relative surface covered by the HepG2 cells in the CQ-treated groups was larger than in the control group (*P* < 0.01).

**Fig. 3:**
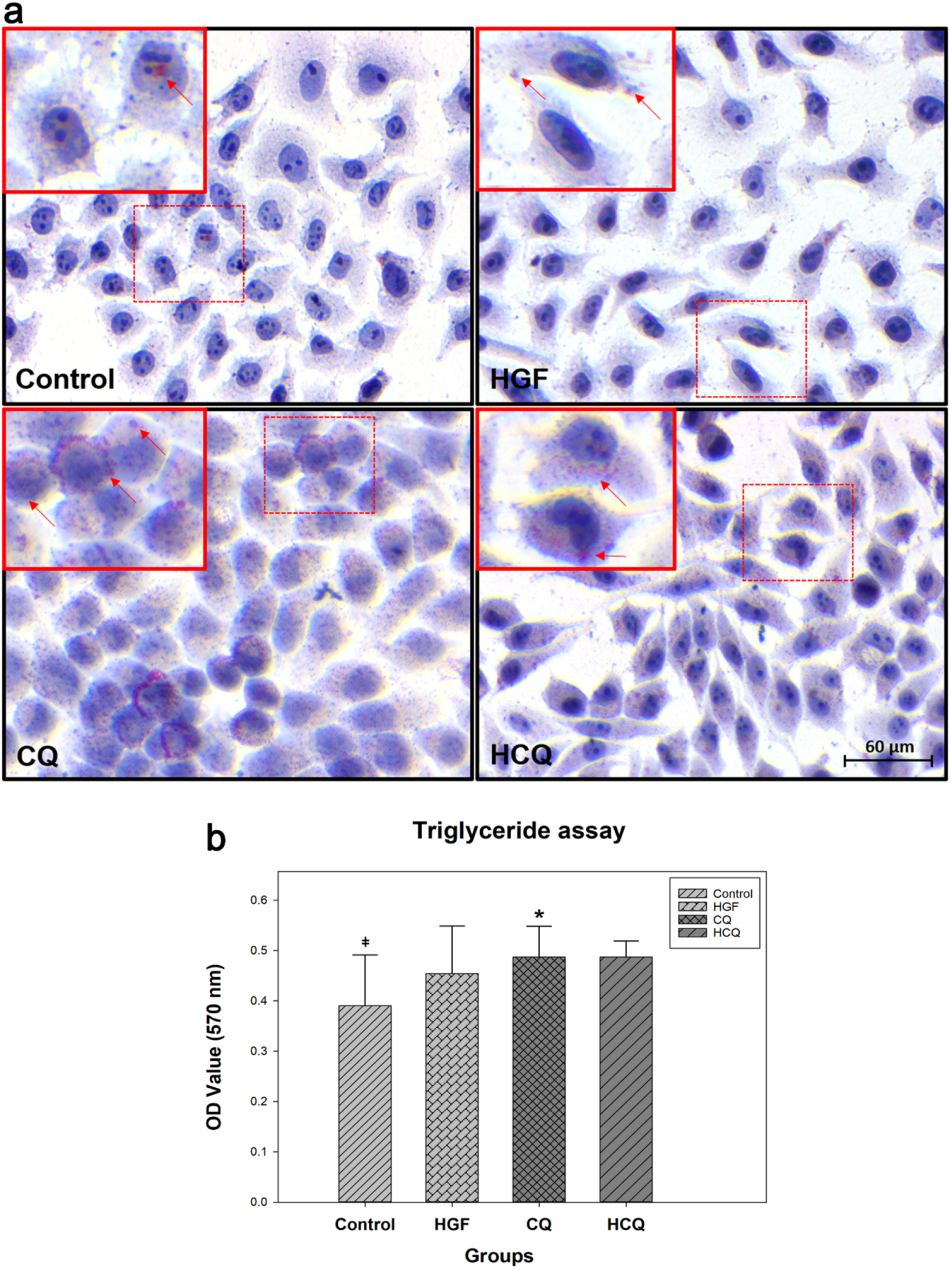
Chloroquine aggravated lipid accumulation in HepG2 cells. **a** HepG2 cells were treated with different drugs for 24 h and the lipid droplets were stained by Oil Red O staining (Magnification: 400X); **b** Intracellular TG content was measured by Triglyceride quantification assay. (*represents significant difference compared to control, # represents significant difference compared to HGF, ǂ represents significant difference compared to HCQ)

Oil Red O staining (Fig. 3a) was used to assess the nature of the intracytoplasmic vesicles. Since the vesicles were stained, we concluded that the CQ-treated cells accumulated more fat than the cells in the control group. The number of lipid droplets in the HepG2 cells treated either with CQ or HCQ groups was substantially higher compared to the control group. There was no obvious visible difference in lipid droplets when comparing control and HGF group.

For further confirmation, we quantified triglyceride in the cells. At the end of the culture period, HepG2 cells were harvested (1 × 10^7^/group) and lysed for triglyceride (TG) quantification assay. The results of TG assay (Fig. 3b) showed that intracellular triglyceride levels in the CQ and HCQ groups increased significantly by approximately 25% compared to the control group (*P* < 0.05).

### Chloroquine significantly reduced ATP synthesis in HepG2 cells

**Fig. 4:**
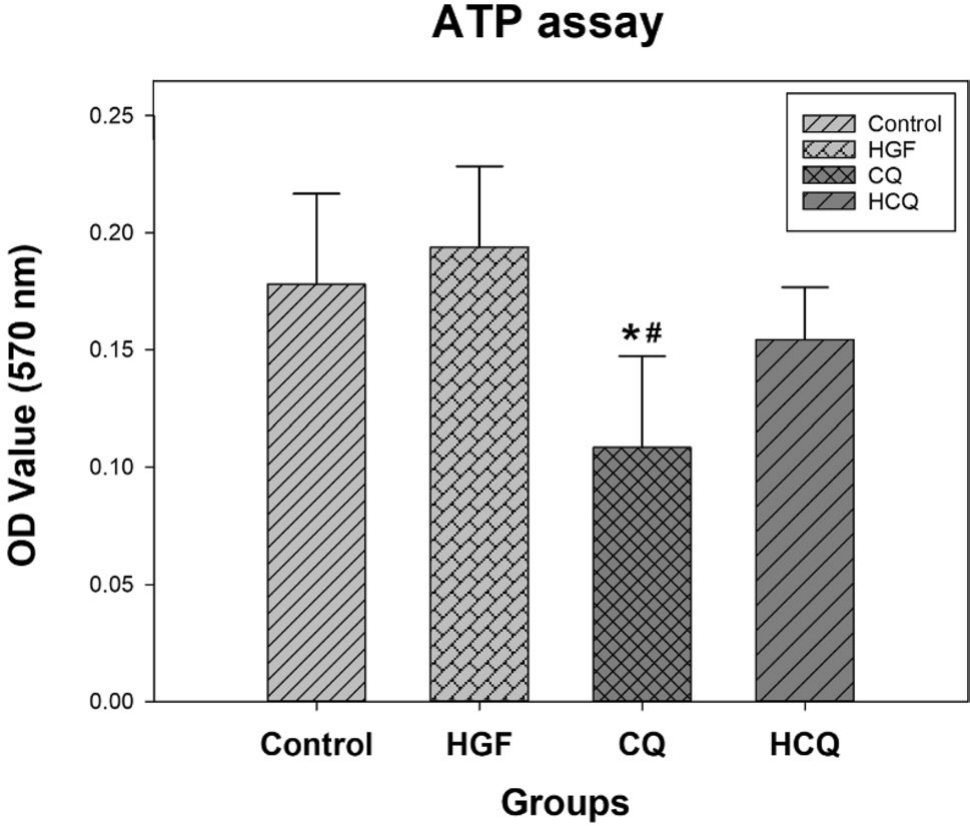
Chloroquine reduced intracellular ATP synthesis. HepG2 cells were treated with different drugs for 24 h and the cellular ATP content was determined by ATP detection assay. (*represents significant difference compared to control, # represents significant difference compared to HGF, ǂ represents significant difference compared to HCQ)

HepG2 cells (3 × 10^6^/group) were harvested and lysed as needed for the ATP assay. CQ treatment resulted in significantly lower ATP content compared to the control group (*P* < 0.05), suggesting inhibition of cellular energy synthesis and proliferation (Fig. 4). In detail, the ATP content of the CQ-treated HepG2 cells decreased by approximately 39%. However, treating the cells only with HGF increased the ATP content slightly, but not significantly. Similarly, subjecting the cells to both HGF and CQ resulted in a slightly lower ATP content, again without reaching statistical significance. Compared with the HGF group, the cellular ATP content in the HCQ group decreased by about 20%, but the difference did not reach significance.

### Chloroquine blocked autophagy in HepG2 cells

The lipidated LC3, known as LC3-2, is a marker of autophagosomes (Bresciani et al. 2018). p62 is an autophagy adapter that recruits ubiquitinated cargo for autophagic degradation. The degradation of p62 acts as another indicator of autophagy activity as p62 is an autophagy substrate, p62 binds to LC3 and is selectively degraded by autophagy (Aparicio et al. 2019; Bresciani et al. 2018; Yoshii and Mizushima 2017).

**Fig. 5:**
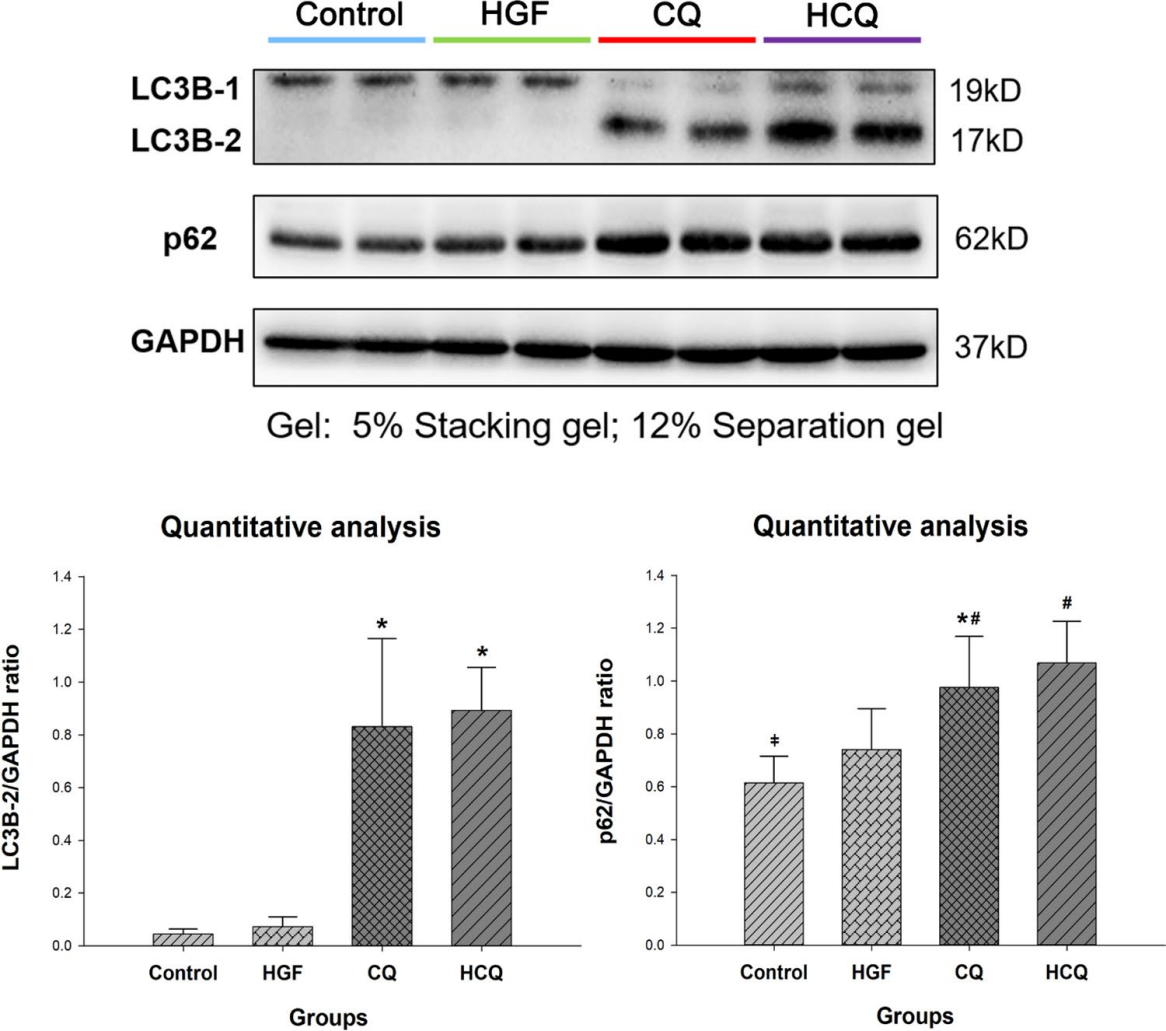
Chloroquine inhibited autophagy in HepG2 cells. HepG2 cells were treated with different drugs for 24 h and protein expression of LC3B and p62 was assessed by western blot, GAPDH was used as control. (*represents significant difference compared to control, # represents significant difference compared to HGF, ǂ represents significant difference compared to HCQ)

Western blot results showed a significant accumulation of LC3B-2 in CQ and HCQ treated cells compared with the control group (Fig. 5), along with a significant increase in p62 protein expression (*P* < 0.01), indicating an inhibition of autophagy. Furthermore, the protein expression of LC3B-2 was also slightly, but not significantly higher in the cells of the HCQ group compared to the CQ group.

### Chloroquine down-regulated autophagic flux in HepG2 cells

**Fig. 6:**
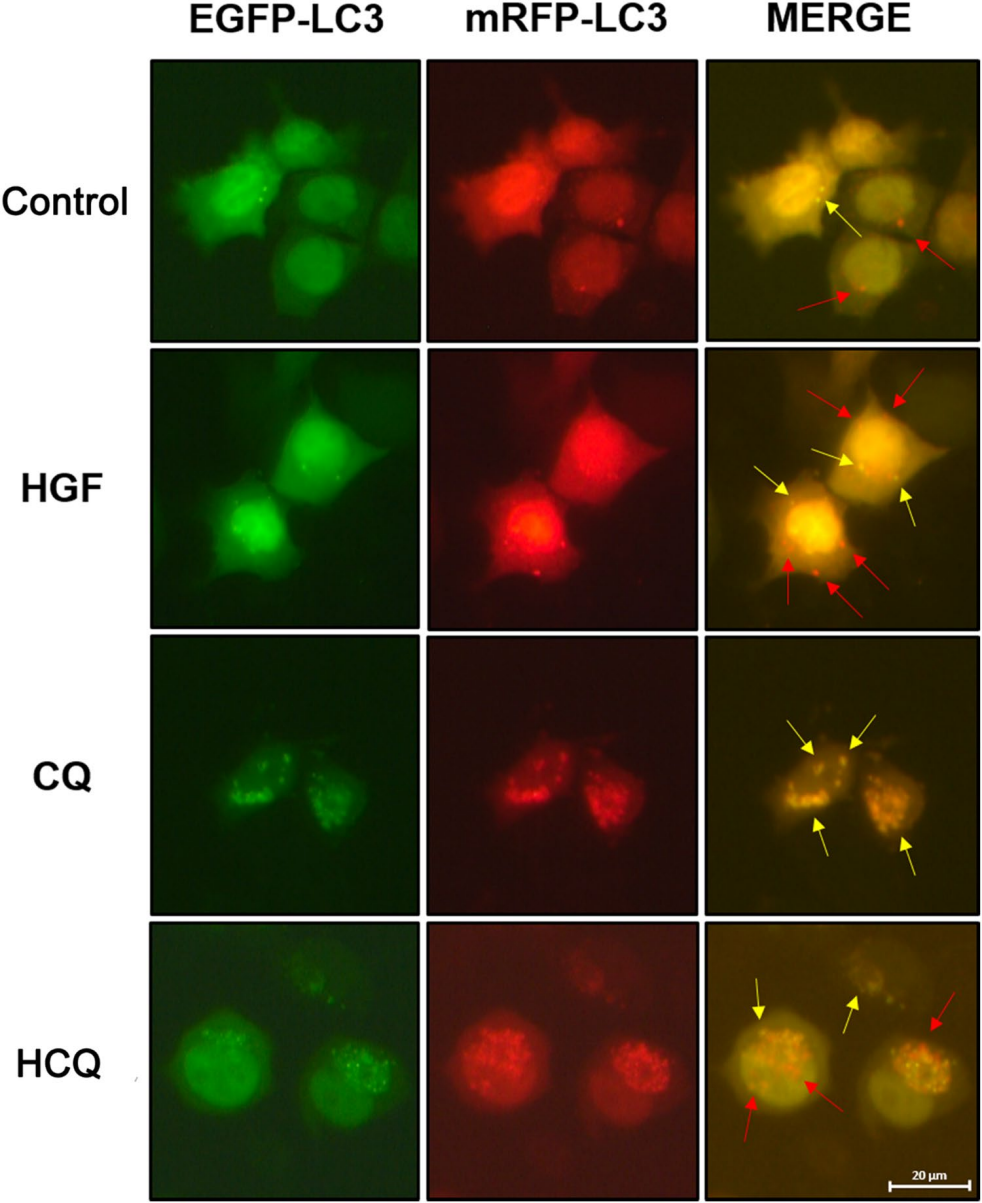
Chloroquine reduced autophagic flux in HepG2 cells. HepG2 cells were treated with different drugs for 24 h and the autophagic flux was detected by mRFP-EGFP-LC3 fluorescence assay (Magnification: 400X). The yellow dots in the images reflect autophagosomes (Indicated by yellow arrow) and the red dots reflect autolysosomes (Indicated by red arrow)

To further verify the effect of chloroquine on autophagy in HepG2 cells, using the mRFP-EGFP-LC3 fluorescence assay, a substantial decrease in autophagic flux in CQ-treated HepG2 cells became obvious (Fig. 6). EGFP and mRFP were used to label and track LC3. The quenching of EGFP indicates the fusion of autophagosome and lysosome to form autolysosome as GFP is sensitive to acid. When autophagosomes and lysosomes are fused, the GFP fluorescence is quenched, and only RFP can be detected at this time. The yellow dots, appearing after the fusion of red and green channel images, reflect autophagosomes (RFP + GFP), the red dots reflect autolysosomes (RFP).

The relative number of red dots was substantially decreased in CQ-treated HepG2 cells compared to control group, in contrast to a visible increase in the number of yellow dots, indicating that the cellular autophagic flux was blocked. There was no obvious difference in autophagic flux between the cells in HGF group and control group. Compared with the CQ group, the autophagic flux in the cells of HCQ group seems to be restored to some extent.

## Discussion

Currently, the commonly used drug for systemic therapy in case of hepatocellular carcinoma is sorafenib. Sorafenib is a multiple tyrosine kinase inhibitor. The drug is inhibiting tumor cell proliferation and is blocking tumor angiogenesis via targeting kinases in various oncogenic signaling pathways such as Raf-1, Vascular endothelial growth factor receptor (VEGFR) and Platelet-derived growth factor receptor (PDGFR) (Gong et al. 2017; Hasskarl 2014; Liu et al. 2006). However, sorafenib has its own drug-related adverse effects possibly affecting the quality of life substantially. As reported by Takeda et al. and others, the patients experienced side effects such as heart failure, acute interstitial pneumonia, and hypertension while taking sorafenib and had to reduce or stop taking the drug (Takeda et al. 2012; Wu and Shemisa 2017; Wu et al. 2008).

Therefore, it is interesting to consider other drugs potentially suitable to complement the therapy with TKI. Modulators of autophagy, one of the hotspots of current medical research, might be of interest for this purpose.

Autophagy has a pro-survival activity allowing cells to accommodate cellular stressors such as hypoxia, oxidative stress and nutrient starvation (He and Klionsky 2009). It plays a crucial role in hepatic physiological processes such as lipid degradation and gluconeogenesis (Ezaki et al. 2011; Singh et al. 2009). HCCs proliferation requires abundant energy and cellular substances for DNA replication and cell division (Alexandrino et al. 2018). HCCs are highly dependent on lipid metabolism as an energy source to sustain rapid cell proliferation (Hayes et al. 2019). In HCCs as in other cells, autophagy is an essential process to release free fatty acids into the cytoplasm. Therefore, autophagy may provide the energy required for HCCs by degrading intracellular lipids to FFAs, which undergo β-Oxidation to generate ATP (Liu and Czaja 2013).

In contrast, blocking autophagy may reduce the energy-producing capacity of the tumor cells, thereby reducing their proliferation rate. An increasing number of drugs inhibiting autophagy are nowadays available, one of them being chloroquine. Chloroquine inhibits the completion of the autophagy process mainly by preventing the fusion of autophagosomes and lysosomes (Mauthe et al. 2018). Our study showed that treating HepG2 cells with the autophagy inhibitor chloroquine led to intracellular lipid accumulation suggesting an impairment of lipid degradation. Intracellular lipid accumulation was associated with a significant downregulation of ATP synthesis. In return, the lack of energy in form of ATP may explain the inhibition of cell proliferation.

(Shimizu et al. 2012) showed that inhibition of autophagy using either an ATG7 knockdown or chloroquine enhanced the antitumor effect of sorafenib in hepatocellular carcinoma. First, they observed that treatment with sorafenib only activated the autophagic flux in HCCs, which theoretically may give a survival advantage to HCCs. However, in a second step, they demonstrated that sorafenib decreased cell viability and induced apoptosis in HCCs. More importantly, in a third step, they observed that inhibition of autophagy by knockdown of ATG7 in the cells significantly enhanced sorafenib-induced apoptosis and further reduced the viability of HCCs. In their fourth and last step, they evidenced that inhibition of autophagy by chloroquine also increased the sensitivity of HCCs to sorafenib, as evidenced by a further decrease in cell activity and an increase in apoptosis. Similar results were also observed in the study of Shi et al. in HCCs (Shi et al. 2011), but also by others using tumor cell lines of different origin, see Table 1.

**Table 1:** Combining sorafenib with autophagy inhibitors like chloroquine enhances the effect of targeted therapy with tyrosine kinase inhibitor on tumor cells of different origin

Author year	Tumor type	Experimental type	Autophagy inhibition	Targeted process
(Shi et al. 2011)	Hepatocellular carcinoma	In vitro and vivo	Chloroquine; 3-Methyladenine; ATG5-KD*	Growth inhibition; Pro-apoptosis
(Shimizu et al. 2012)	Hepatocellular carcnoma	In vitro and vivo	Chloroquine; ATG7-KD	Pro-apoptosis
(Zheng et al. 2015)	Renal cell carcinoma	In vitro	Chloroquine; Bafilomycin-A1; 3-Methyladenine; MiRNA-30a; Beclin-1-KD	Pro-apoptosis
(Liu et al. 2016)	Glioblastoma	In vitro and vivo	Chloroquine	Anti-proliferation; Pro-apoptosis
(Rong et al. 2017)	Hepatocellular carcinoma	In vitro	Chloroquine; 3-Methyladenine	Pro-cell death
(Yi et al. 2018)	Thyroid cancer	In vitro and vivo	Chloroquine; ATG5-KD	Anti-proliferation; Pro-apoptosis

*KD knockdown

Our results further explain why adding autophagy inhibitors like chloroquine to treatment with TKI may increase the therapeutic efficacy. We demonstrated that chloroquine is affecting cellular energy metabolism by reducing the intracellular ATP levels. This in return, inhibits proliferation, especially of the energy-demanding tumor cells.

Combining TKI with chloroquine might, therefore, result in three beneficial effects: increased efficiency, reduced dose, lower treatment costs.

As outlined in Table 1, the combination of TKI with chloroquine sensitizes tumor cells for sorafenib by interfering with two relevant cellular processes: proliferation (inhibition) and apoptosis (upregulation).

Furthermore, combination treatment might allow a dose reduction to reduce the number and severity of adverse effects compared to the use of TKI alone. However, this needs to be demonstrated. At this point, it was experimentally shown by (Shi et al. 2011) that the treatment of sorafenib combined with chloroquine in nude mice inoculated with HCCs did not cause any substantial hepatotoxicity. Besides, the clinical study by (Tovoli et al. 2019) showed that the use of a lower median daily dose of sorafenib reduced the rate of permanent discontinuation due to adverse events. More importantly, in their experiment, the use of a lower median daily dose of sorafenib significantly prolonged the median treatment duration with a trend towards a higher cumulative dose and improved overall survival in patients with hepatocellular carcinoma. Therefore, the combination of chloroquine and low-dose sorafenib may further enhance tumor suppression and prolong the duration of treatment.

In addition, combination treatment might lower the therapy costs. The high price of the drug may represent a substantial burden for the patients if not covered by the respective health system. In detail, chloroquine has an economical price compared to sorafenib (Chloroquine 250 mg is about $ 8/unit and Sorafenib 200 mg is about $ 181/unit (Drugs.com 2021a, b). Chloroquine is now widely used clinically to treat a variety of immune diseases and its safety has been tested in both acute and chronic administration (Savarino et al. 2003). Therefore, reducing the dose in an eventual combination therapy with another safe, effective and affordable drug like chloroquine might be of advantage.

However, the molecular pathways involved in the regulation of energy metabolism through chloroquine in HCCs are still unclear. Therefore, further in-depth exploration of pathways involved in the inhibition of cellular ATP production by chloroquine would lead to a better understanding of energy metabolism in hepatic tumor cells. A systematic study is needed addressing all potentially involved pathways such as p53 and Wingless-related integration site (Wnt) (Mo et al. 2019; Puzio-Kuter 2011).

Besides, the role of regulating autophagy in inhibiting tumor progression deserves further investigation e.g. (a) Do tumor cells become resistant to chloroquine under long-term chloroquine intervention? (b) Does autophagy of tumor cells provide a facilitative effect on the development of sorafenib resistance? Attempts to establish a chloroquine or sorafenib-resistant HepG2 cell line will help to further explore the above questions.

**Fig. 7:**
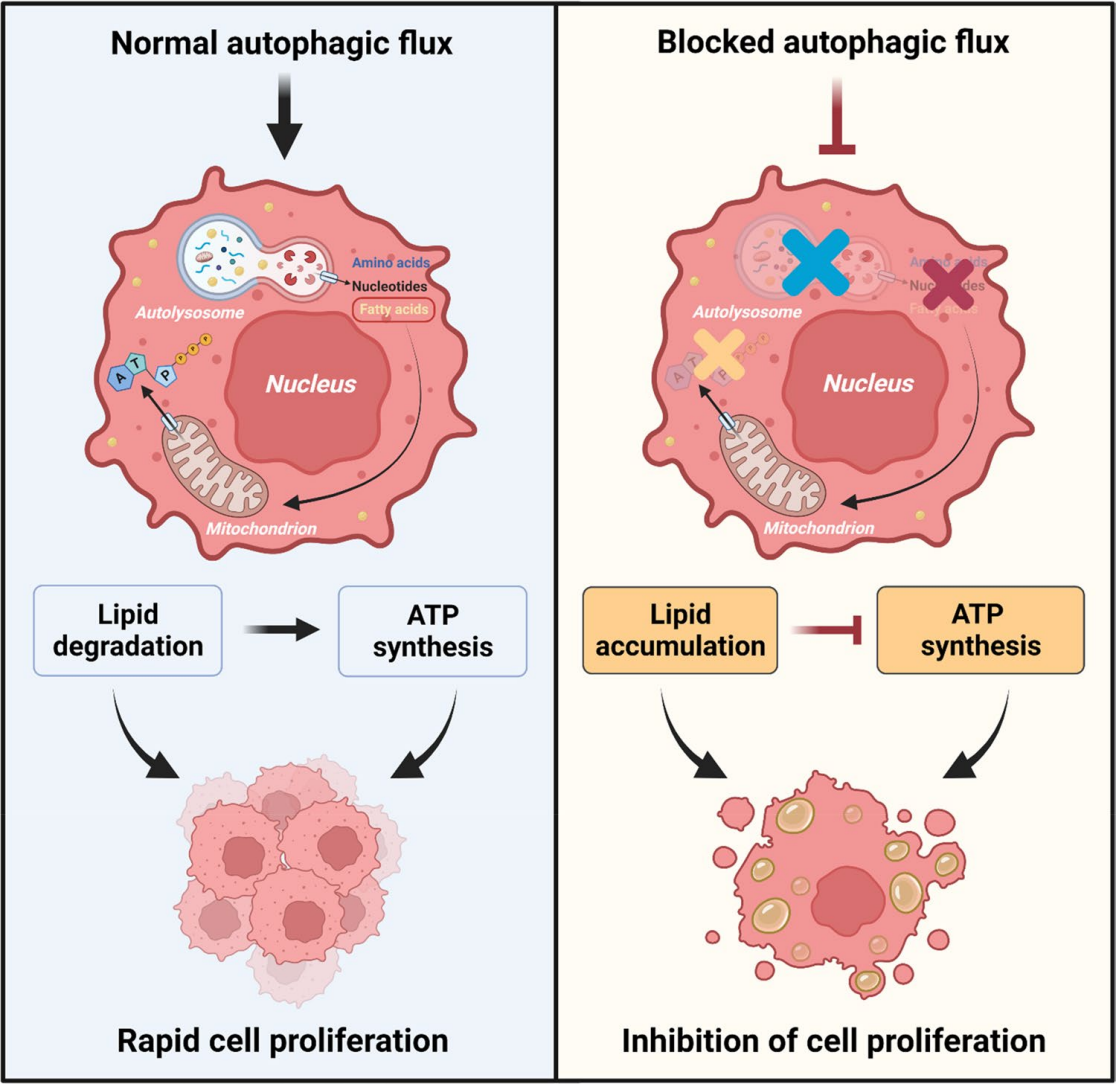
Chloroquine aggravates intracellular lipid accumulation and reduces energy synthesis in HepG2 cells via blocking autophagy to inhibit cell proliferation

In summary, our results suggest that treatment with the autophagy inhibitor chloroquine caused lipid accumulation in HepG2 cells thereby reducing intracellular ATP synthesis (Fig. 7). Both together contributed to the inhibition of cell proliferation. It is of importance to further explore the underlying molecular mechanism in more depth using hepatocellular tumor cells as well as mature isolated hepatocytes. Doing so might turn out in a next step on the road to integration in a clinical concept.

## Data Availability

Data are available from the corresponding author on reasonable request.

## Abbreviations

AMPK: AMP-activated protein kinase
AMP: Adenosine monophosphate
ATG5: Autophagy-related 5
ATG7: Autophagy-related 7
ATP: Adenosine triphosphate
BCA: Bicinchoninic acid assay
CCK-8: Cell counting kit 8
CQ: Chloroquine
DMEM: Dulbecco’s modified eagle medium
EGFP: Enhanced green fluorescent protein
FBS: Fetal bovine serum
FFA: Free fatty acid
GAPDH: Glyceraldehyde-3-phosphate dehydrogenase
HCC: Hepatoma carcinoma cell
HCQ: Hepatocyte growth factor and chloroquine
HGF: Hepatocyte growth factor
KD: Knockdown
LB: Lysogeny broth
LC3: Microtubule-associated protein 1A/1B-light chain 3
mRFP: Monomeric red fluorescent protein
OD: Optical density
PDGFR: Platelet-derived growth factor receptor
P/S: Penicillin and streptomycin
PVDF: Polyvinylidene fluoride
RIPA: Radioimmunoprecipitation assay
RT: Room temperature
S6K1: Ribosomal protein S6 kinase beta-1
TBST: Tris-buffered saline with tween 20
TG: Triglyceride
TKI: Tyrosine kinase inhibitor
ULK1: Unc-51-like autophagy activating kinase 1
VEGFR: Vascular endothelial growth factor receptor
Wnt: Wingless-related integration site
